# Prediction of gestational diabetes mellitus in Asian women using machine learning algorithms

**DOI:** 10.1038/s41598-023-39680-8

**Published:** 2023-08-16

**Authors:** Byung Soo Kang, Seon Ui Lee, Subeen Hong, Sae Kyung Choi, Jae Eun Shin, Jeong Ha Wie, Yun Sung Jo, Yeon Hee Kim, Kicheol Kil, Yoo Hyun Chung, Kyunghoon Jung, Hanul Hong, In Yang Park, Hyun Sun Ko

**Affiliations:** 1 Department of Obstetrics and Gynecology, Seoul St. Mary’s Hospital, College of Medicine, The Catholic University of Korea, Seoul, Korea; 2 Department of Obstetrics and Gynecology, St. Vincent’s Hospital, College of Medicine, The Catholic University of Korea, Seoul, Korea; 3 Department of Obstetrics and Gynecology, Incheon St. Mary’s Hospital, College of Medicine, The Catholic University of Korea, Seoul, Korea; 4 Department of Obstetrics and Gynecology, Bucheon St. Mary’s Hospital, College of Medicine, The Catholic University of Korea, Seoul, Korea; 5 Department of Obstetrics and Gynecology, Eunpyeong St. Mary’s Hospital, College of Medicine, The Catholic University of Korea, Seoul, Korea; 6 Department of Obstetrics and Gynecology, Uijeongbu St. Mary’s Hospital,, College of Medicine, The Catholic University of Korea, Seoul, Korea; 7 Department of Obstetrics and Gynecology, Yeouido St. Mary’s Hospital, College of Medicine, The Catholic University of Korea, Seoul, Korea; 8 Department of Obstetrics and Gynecology, Daejeon St. Mary’s Hospital, College of Medicine, The Catholic University of Korea, Seoul, Korea; 9 Innerwave Co., Ltd, Seoul, Korea

**Keywords:** Gestational diabetes, Risk factors, Computer science

## Abstract

This study developed a machine learning algorithm to predict gestational diabetes mellitus (GDM) using retrospective data from 34,387 pregnancies in multi-centers of South Korea. Variables were collected at baseline, E0 (until 10 weeks’ gestation), E1 (11–13 weeks’ gestation) and M1 (14–24 weeks’ gestation). The data set was randomly divided into training and test sets (7:3 ratio) to compare the performances of light gradient boosting machine (LGBM) and extreme gradient boosting (XGBoost) algorithms, with a full set of variables (original). A prediction model with the whole cohort achieved area under the receiver operating characteristics curve (AUC) and area under the precision-recall curve (AUPR) values of 0.711 and 0.246 at baseline, 0.720 and 0.256 at E0, 0.721 and 0.262 at E1, and 0.804 and 0.442 at M1, respectively. Then comparison of three models with different variable sets were performed: [a] variables from clinical guidelines; [b] selected variables from Shapley additive explanations (SHAP) values; and [c] Boruta algorithms. Based on model [c] with the least variables and similar or better performance than the other models, simple questionnaires were developed. The combined use of maternal factors and laboratory data could effectively predict individual risk of GDM using a machine learning model.

## Introduction

Gestational diabetes mellitus (GDM) is one of the most common medical complications of pregnancy, and its prevalence has increased significantly worldwide^1^. The pooled global standardized prevalence of GDM was 14.0% in 2022^2^, and it is expected to continue to increase in the future. However, the prevalence of GDM is also highly variable due to racial, ethnic, and population diversity, as well as heterogeneous screening tools and diagnostic strategies^3^. Several studies have found that Asian women have a higher risk of GDM than other ethnic groups, despite having a relatively lower body mass index (BMI)^4,5^. In a meta-analysis of Eastern and Southeastern Asian populations, the prevalence of GDM was found to be 10.1%^6^ to 20%^2,7^. The prevalence of GDM in South Korea was reported to be 8% to 11.1% in 2012–2016 and it continues to increase^3,6^.

Hyperglycemia in pregnancy can be harmful to the fetus and lead to several neonatal complications^8^. GDM is also known to be associated with an increased risk of type II diabetes and cardiovascular disease in the mother in later life^9,10^ and obesity, diabetes, and metabolic syndrome in the childhood period of offspring^11^.

Although there is no clear consensus on the best screening method for GDM^12^, early detection may prevent or reduce the risk of adverse pregnancy outcomes^13,14^. A multicenter Randomized Controlled Trial is underway to evaluate the benefits of early screening and management of GDM; and it suggests that early screening may help improve prognosis^15^. Although early pregnancy risk stratification may help to promote lifestyle modification aimed at reducing the risk of GDM^16^, there is no consensus on the optimal screening algorithms. Further research is needed to determine when screening tests should be performed, which tests are the most accurate, and which individuals should be tested^17^.

Recently, various clinical prediction models have been developed to identify women at increased risk of GDM^18,19^, and several machine learning methods have demonstrated robust self-learning capabilities with improved GDM prediction accuracy^20–22^. Therefore, this study aimed to develop a machine learning algorithm for prediction of GDM in Asian women according to gestational period that can be applied to Korean women to calculate individualized risk of GDM.

## Methods

### Data

Data were extracted from the perinatal database for women who delivered between January 2009 and December 2020 at seven hospitals in four areas of South Korea, under the authority of The Catholic University of Korea. Data on maternal demographic characteristics, body mass index (BMI), blood pressure (BP) measurements, blood and urine laboratory tests, diagnoses recorded by physicians, and medications prescribed were collected from the hospitals’ databases via electronic medical records. Data were confirmed, and missing data were abstracted from chart reviews by two obstetricians (J.H.W. and H.S.K.). The Institutional Review Board of The Catholic University of Korea approved the present study (XC20WIDI0103). Since this study analyzed retrospective cohort data, which were anonymized, informed consent was waived by the Institutional Review Board of The Catholic University of Korea.

### Study population and outcome definition

In medical centers of The Catholic University of Korea, GDM is diagnosed by a two-step procedure performed routinely for all women at 24–28 weeks of pregnancy, in accordance with NIH guidelines^23,24^. In the first step, a 1-h, 50-g, glucose challenge test (GCT) is performed; women with glucose levels > 200 mg/dl receive a GDM diagnosis. Women with a GCT value > 140 mg/dl are referred to a second step, in which an additional 100-g, 3-h oral glucose tolerance test (OGTT) is performed. Women with two glucose measurements above the Carpenter and Coustan criteria (thresholds of 95, 180, 155, and 140 mg/dl) under fasting conditions (zero), 1, 2, and 3 h after glucose intake, respectively, also receive a GDM diagnosis^25^. In women with risk factors such as a family history of diabetes or history of GDM in a previous pregnancy, GDM screening is sometimes performed in the first trimester. In some women with a 50-g GCT value ≤ 140 mg/dl, at 24–29 weeks’ gestation, but with a fetus suspected to be large for gestational age, (> 90th percentile) during the second or third trimester, a 100-g OGTT was performed and GDM was diagnosed using the Carpenter and Coustan criteria. We also included pregnancies with GDM based on the ICD code during pregnancy (ICD9 codes in O24.4 or O24.9), after excluding women with a prepregnancy record of diabetes, defined as any of the ICD9 codes in O24.0–24.3, or E12–14, or prescription codes for insulin or other medication for diabetes.

### Machine learning

#### Data preparation

We followed the guidelines for the Transparent Reporting of a Multivariable Prediction Model for Individual Prognosis or Diagnosis for Establishing Prediction models^26^. Based on these guidelines, all anonymized data from 34,387 subjects were included in the data set used for this study. The subjects were divided into two groups based on their parity status: nulliparity (n = 18,023) and multiparity (n = 16,184). To ensure a balanced distribution of the target variable, a 7:3 stratified split was performed to divide the data into training and test sets for each of the three cohorts: the whole cohort, nulliparity cohort, and multiparity cohort. Each cohort of training and test data was analyzed by dividing it into GDM and non-GDM groups.

#### Variables used to develop the gestational diabetes prediction models

Four sets of variables were used to develop the GDM prediction models, including variables from different time periods: (1) baseline variables, which were obtained solely from maternal questionnaires completed during the first hospital visits; (2) early pregnancy (E0) variables, collected from the first visit until 10 weeks of gestation; (3) 1st trimester (E1) variables, collected from 11 to 13 weeks of gestation; and (4) 2nd trimester (M1) variables, collected from 14 to 24 weeks of gestation. The variables included age, parity, underlying diseases, family history, reproductive history, physical examinations, laboratory results, and obstetric histories of the previous pregnancy in parous women. All sets included age, parity, underlying diseases, reproductive history, obstetric histories of the previous pregnancy, physical examination, and family history. The baseline set included physical examinations, but laboratory results were excluded. This resulted in inclusion of 165 variables in the nulliparous women and 199 variables in the parous women. The E0 set included physical examinations and laboratory test results before the first trimester serum screening, which resulted in inclusion of 222 variables in the nulliparous women and 254 variables in the parous women. The E1 set included data from maternal serum Down screening tests of human chorionic gonadotropin (HCG) and pregnancy associated plasma protein-A (PAPP-A), which resulted in inclusion of 224 variables in the nulliparous women and 256 variables in the parous women. The M1 set included the 50-g GCT laboratory test results, which resulted in inclusion of 324 variables in the nulliparous women and 361 variables in the parous women. The whole cohort used the same sets of variables as those used for the multiparity cohort, according to each time point.

### Machine learning algorithms

In this study, two tree-based algorithms were compared: the light gradient boosting machine (LGBM) and extreme gradient boosting (XGBoost) algorithms. Both algorithms implement the gradient boosting method, which is widely recognized as the state of the art for predicting tabular data^27,28^. Additionally, these algorithms have built-in methods for handling missing values, making it possible to use data with missing values in the machine learning process^29^.

### Evaluation of algorithms

To determine the best algorithm to use, the split data set was fed into each algorithm and its performance was evaluated using the metrics of area under the receiver operating characteristic curve (AUC) and area under precision-recall curve (AUPR). The performances of LGBM and XGBoost across the whole data set, nulliparity, and multiparity cohorts, at four different stages (baseline, E0, E1, and M1), were compared and a final algorithm was selected for prediction.

### Model interpretations

In order to explain the output from complex machine learning models, Shapley values were used to indicate how much influence each variable had in determining model output. The Shapley value is the average value of all the contribution of every variables in a coalition, according to the presence or absence of the each variable. We used a SHapley Additive exPlanations (SHAP) values to calculate and visualize the Shapley values of the prediction models^30^.

### Simplified model evaluation and validation

For clinical application, three models were developed with simplified features. Model [a] used variables suggested by the American College of Obstetricians and Gynecologists (ACOG) and the American Diabetes Association (ADA) as GDM risk factors for early pregnancy screening^24,31^. Model [b] used variables with high ranked SHAP values up to 40 variables. Model [c] utilized the Boruta algorithm to select important variables for the machine learning model^32^. The Boruta algorithm is a variable selection algorithm based on the random forest and decision trees. Removing variables that are less relevant to the result than their shadowed variable by a statistical test can reduce the misleading impact of random fluctuations and correlations and reduce the undesirable effect of over-fitting^32,33^. The performance of these three models was evaluated by AUC and AUPR and validated in the test sets. Questionnaires for clinical application were developed based on the model performance and convenience.

### Ethical approval

The Institutional Review Board of The Catholic University of Korea approved the present study (XC20WIDI0103).

## Results

### Data set

**Figure 1:**
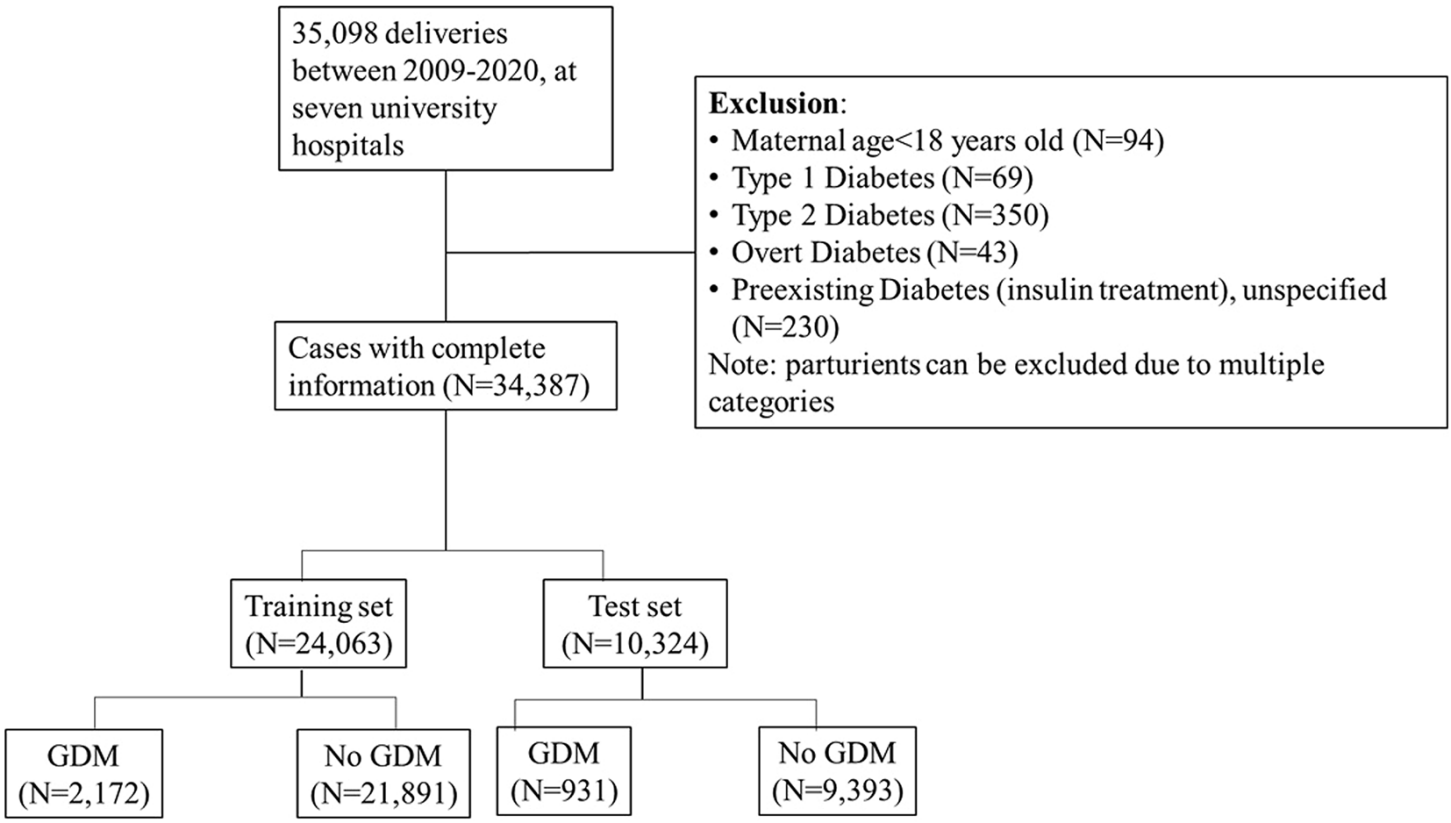
Participant selection process. GDM, gestational diabetes.

The final study cohort included 34,387 pregnancies after subtracting women with exclusion criteria from among 35,098 pregnancies during the study period (Fig. 1). GDM was diagnosed in 3,103 pregnancies (9.02%) in the entire cohort, which were 2,172 and 931 pregnancies in the training and test sets, respectively.

### Algorithm selection

**Figure 2:**
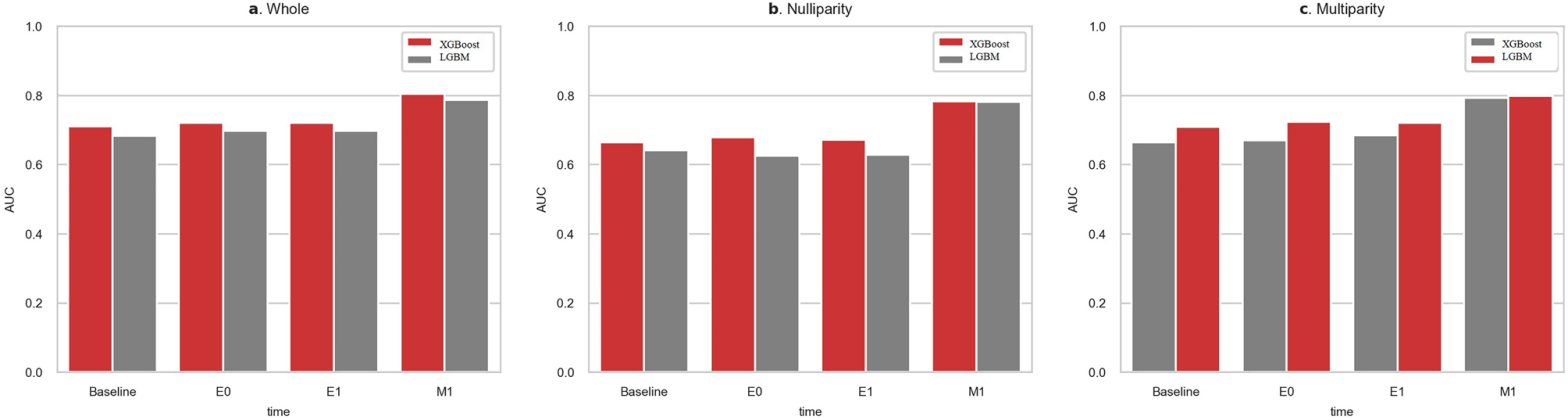
Performance comparison between LGBM and XGBoost algorithms, using original variables. (**a**) AUC performance at each time point in the whole cohort, (**b**) AUC performance at each time point in the nulliparity cohort, (**c**) AUC performance at each time point in the multiparity cohort. LGBM, light gradient boosting machine; XGBoost, extreme gradient boosting.

When all the original variables were used, XGBoost outperformed LGBM in most cohorts and at most time points, except for the E1 time point in the whole and multiparity cohort (Fig. 2). Therefore, XGBoost was selected for subsequent analysis.

#### Predictive model evaluation

**Figure 3:**
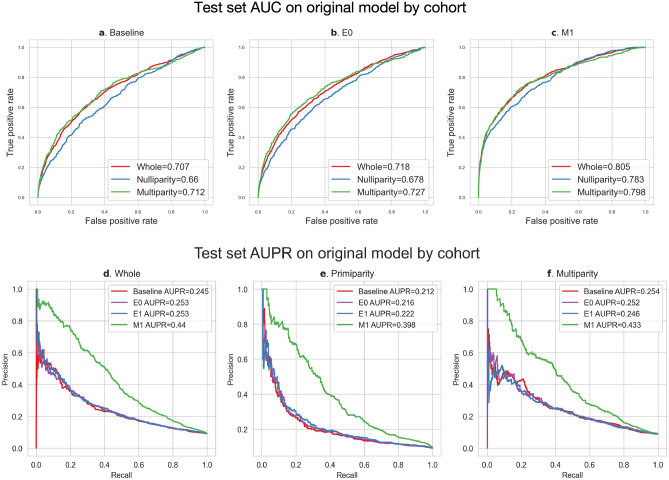
Predictive model evaluation with original variables. (**a**) AUC curves at each time point in the whole cohort, (**b**) AUC curves at each time point in the nulliparity cohort, (**c**) AUC curves at each time point in the multiparity cohort, (**d**) AUPR curves at each time point in the whole cohort, (**e**) AUPR curves at each time point in the nulliparity cohort, (**f**) AUPR curves at each time point in the multiparity cohort.

A prediction model with original variables achieved an AUC of 0.711 at baseline, 0.720 at E0, 0.721 at E1 and 0.804 at M1, and an AUPR of 0.246 at baseline, 0.256 at E0, 0.262 at E1, and 0.442 at M1, for the whole cohort (Fig. 3). In the nulliparous cohort, a prediction model with original variables achieved an AUC of 0.664 at baseline, 0.678 at E0, 0.672 at E1, and 0.782 at M1, and an AUPR of 0.211 at baseline, 0.229 at E0, 0.226 at E1, and 0.413 at M1. In the multiparous cohort, a prediction model with original variables achieved an AUC of 0.708 at baseline, 0.723 at E0, 0.720 at E1, and 0.798 at M1, and an AUPR of 0.260 at baseline, 0.274 at E0, 0.240 at E1, and 0.424 at M1.

**Figure 4:**
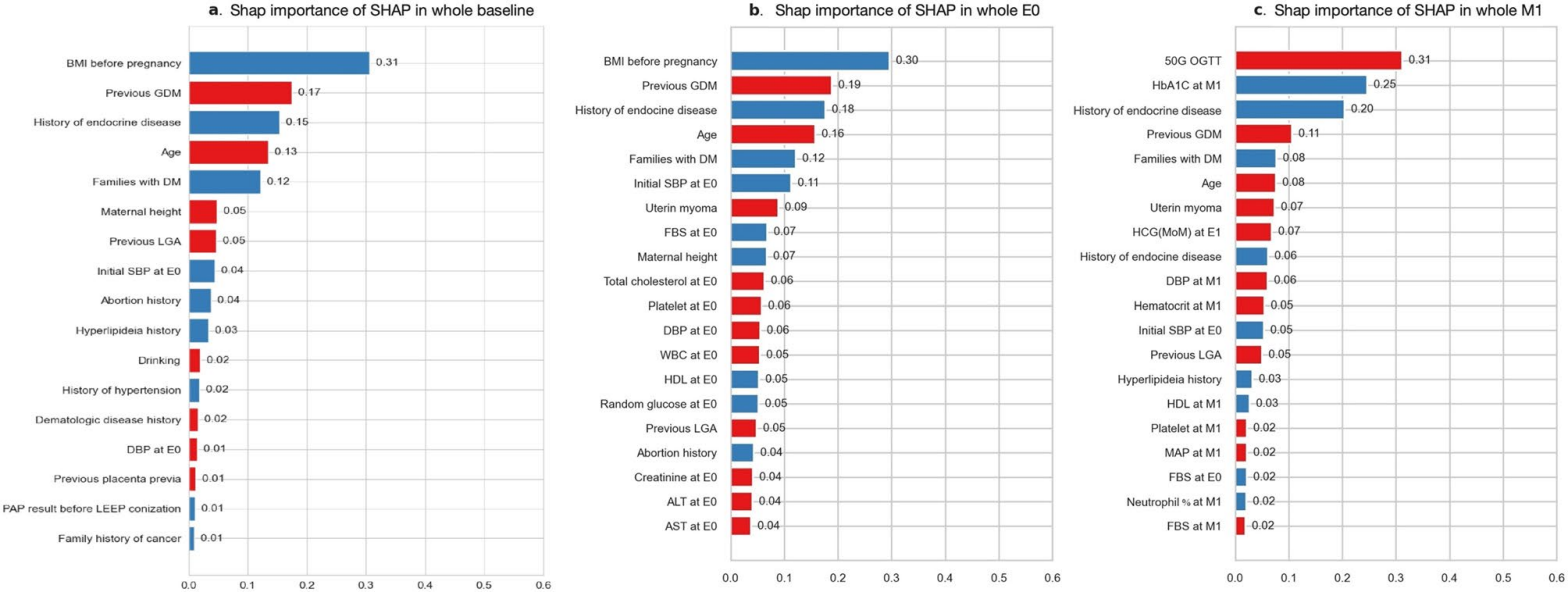
Feature importance of the top 20 contributing features in the whole cohort. Each graph illustrates the importance of the top 20 items among 40 items with a high cut-off value extracted through SHAP importance. The red bar indicates high positive importance, and the blue bar means high negative importance. (**a**) baseline, (**b**) E0 period, (**c**) M1 period. BMI, body mass index (kg/m^2^); DM, diabetes; GDM, gestational diabetes; LGA, large for gestational age; SBP, systolic BP; DBP, diastolic BP; WBC, white blood cell; HDL, high density lipoprotein; ALT, alanine aminotransferase; AST, aspartate aminotransferase; OGTT, oral glucose tolerance test; HbA1C, glycated hemoglobin; FBS, fasting blood sugar; HCG, multiples of median values of human chorionic gonadotropin; MAP, mean arterial pressure.

There was no improvement in predictive power in the E1 period compared to the E0 period, even though more variables were added. Accordingly, the subsequent analysis was conducted at the three time points of baseline, E0, and M1. Feature importance with high SHAP values up to 20 variables in the whole cohort are presented in Fig. 4. BMI before pregnancy, history of previous GDM, history of endocrine disease, maternal age, and family history of DM were identified as the most predictive features at baseline and E0. At M1, the variables that were identified as the most predictive features were the 50-g GCT results, HbA1C levels, BMI before pregnancy, history of previous GDM, and family history of DM.

#### Feature selections for a simplified prediction model

**Figure 5:**
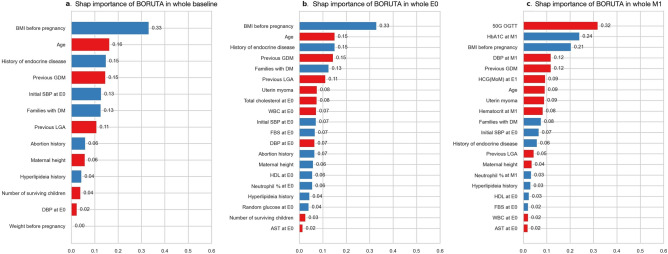
SHAP importance of variables identified by the Boruta algorithm in the whole cohort. (**a**) baseline, (**b**) E0 period, (**c**) M1 period. BMI, body mass index (kg/m^2^); DM, diabetes; GDM, gestational diabetes; LGA, large for gestational age; SBP, systolic BP; DBP, diastolic BP; WBC, white blood cell; HDL, high density lipoprotein; AST, aspartate aminotransferase; OGTT, oral glucose tolerance test; HbA1C, glycated hemoglobin; FBS, fasting blood sugar; HCG, multiples of median values of human chorionic gonadotropin.

For clinical application, we compared the performance of the three models using GDM risk factors recommended by ACOG, top 40 features based on SHAP value, and the Boruta algorithm. We evaluated the performance of each model and compared the results to determine which features and models yielded the best performance for GDM prediction. GDM risk factors recommended by ACOG include being overweight or obese, being physically inactive, having GDM in a previous pregnancy, having a very large baby (9 pounds or more) in a previous pregnancy, high BP, a history of heart disease, and polycystic ovary syndrome (PCOS)^24^. The top 40 variables with the highest SHAP values were identified for each cohort (Fig. 4 and Supplementary Fig. S1), and variables were also determined using the Boruta algorithm (Fig. 5 and Supplementary Fig. S2). The Boruta algorithm selected similar features to the top 40 features identified by SHAP values, but the number of features determined by the Boruta algorithm was 10 at baseline and 20 at E0 and M1 (Figs. 4 and 5). The minimum and maximum SHAP importance of variables identified by the Boruta algorithm are provided in Supplementary Table S3, and the SHAP summary plots are shown in Supplementaty Fig. S4.

#### Performance evaluation of simplified models by feature selection methods

At baseline, the model with GDM risk factors determined by ACOG displayed the lowest performance in the whole, nulliparity, and multiparity cohorts. The performance of the models using SHAP values and the Boruta algorithm were similar at each time point (Table 1). Consequently, we developed simplified 12-point (baseline) and 20-point(E0) and 18-point (M1) questionnaires that corresponds to the variables identified by the Boruta algorithm for clinical use (Table 2).

**Table 1:** Comparison of model performance by feature selection.

Algorithm	Cohort	Time	Training set		Test set	
			AUC	AUPR	AUC	AUPR
Original	Whole	Baseline	0.725	0.26	0.711	0.246
		E0	0.813	0.438	0.72	0.256
		E1	0.817	0.456	0.721	0.262
		M1	0.853	0.552	0.804	0.442
	Nulliparity	Baseline	0.722	0.261	0.664	0.211
		E0	0.788	0.377	0.678	0.229
		E1	0.849	0.541	0.672	0.226
		M1	0.879	0.596	0.782	0.413
	Multiparity	Baseline	0.752	0.297	0.708	0.26
		E0	0.818	0.422	0.723	0.274
		E1	0.884	0.645	0.72	0.24
		M1	0.911	0.705	0.798	0.424
Model [a]						
GDM risk factors by ACOG	Whole	Baseline	0.821	0.451	0.626	0.167
	Nulliparity	Baseline	0.866	0.542	0.595	0.166
	Multiparity	Baseline	0.899	0.635	0.674	0.202
Model [b]						
SHAP value*	Whole	Baseline	0.725	0.26	0.709	0.245
		E0	0.809	0.424	0.714	0.247
		M1	0.85	0.541	0.802	0.441
	Nulliparity	Baseline	0.721	0.257	0.66	0.211
		E0	0.791	0.402	0.678	0.225
		M1	0.877	0.606	0.784	0.403
	Multiparity	Baseline	0.754	0.301	0.711	0.259
		E0	0.818	0.45	0.718	0.267
		M1	0.903	0.684	0.794	0.429
Model [c]						
Boruta** algorithm	Whole	Baseline	0.726	0.251	0.713	0.247
		E0	0.742	0.276	0.71	0.247
		M1	0.879	0.62	0.8	0.434
	Nulliparity	Baseline	0.689	0.222	0.658	0.212
		E0	0.748	0.309	0.675	0.215
		M1	0.958	0.838	0.776	0.389
	Multiparity	Baseline	0.778	0.39	0.712	0.241
		E0	0.888	0.66	0.704	0.238
		M1	0.861	0.552	0.789	0.419

ACOG: the American College of Obstetricians and Gynecologists. *SHAP: Using the 40 top columns based on SHAP value. **Boruta: Only columns extracted as a result of applying the Boruta algorithm to all columns per cohort were used. Union of variables of each time point and each cohort extracted through the Boruta algorithm.

**Table 2:** Simplified questionnaires for clinical application.

**Baseline check (first visit)**
Please complete:
Age __________ years old
Height __________ cm
Weight before pregnant ____________ kg
Current weight ____________ kg
Blood pressure: SBP __________ / DBP __________mmHg
Please check as applicable
History of abortion: Yes / No
Have you ever been diagnosed with endocrine disease? Yes / No
Have you ever been diagnosed with hyperlipidemia? Yes / No
Has anyone in your family been diagnosed with diabetes? Yes / No
Please complete / check only if you have given birth previously
How many children do you have? ________
Have you ever been diagnosed with GDM in a previous pregnancy? Yes / No
Was your baby a large for gestational age in the last pregnancy? Yes / No
**E0 : Early pregnancy, until 10 weeks gestation**
To be completed by the clinician
Uterine myoma: Yes / No
WBC count: ____________10^9^/L
Neutrophil: _____________%
Fasting blood sugar (FBS): ____________ mg/dl
Random glucose: _____________ mg/dl
Total cholesterol: _____________ mg/dl
High density lipoprotein cholesterol: _____________ mg/dl
Aspartate aminotransferase (AST): _____________ U/L
**M1 : Second trimester, from 14 to 24 weeks gestation**
To be completed by the clinician
Blood pressure: SBP _________/ DBP __________mmHg
50G OGTT: _____________ mg/dl
HbA1C: _____________ %
Hematocrit: _____________ %
Neutrophil: _____________ %
HCG _____________ Multiples of Median (MoM) at E1

## Discussion

### Principal findings

In this study, we developed a machine learning algorithm for prediction of GDM in Asian women, especially Korean women, according to gestational period. The XGBoost algorithm displayed better performance than the LGBM algorithm in most cohorts and at most time points. After analyzing the performance of the machine learning model with original variables, we developed three models with simplified features for clinical application. At baseline, the model with GDM risk factors recommended by ACOG displayed the lowest performance for the whole cohort and subcohorts. Although the performance of the models employing SHAP values and the Boruta algorithm was similar at each time point, the model with variables determined by the Boruta algorithm was selected for clinical application, because the number of features was less than in the model using SHAP values. Finally, we developed questionnaires for clinical application at baseline, at E0, and at M1.

Because the first trimester maternal serum aneuploidy screening markers, PAPP-A and HCG, which were added as variables in E1, did not improve the prediction performance for GDM, we set the three time points for prediction as baseline, E0, and M1. As expected, the prediction performance for GDM in the M1 period, which included the results of the 50-g OGTT and HbA1C levels, displayed significant improvement, with the highest SHAP importance of these two variables. Measurement of HbA1C is not included in routine screening tests during pregnancy. However, in South Korea abdominal obesity, assessed using waist circumference in women of 20–49 years old, has increased from 9% in 2013 to 12.2% in 2021^34^. In 2020, the prevalence of diabetes and prediabetes was reported as 14.3% and 22.5% in women of 30–49 years old^35^. Although the high SHAP importance of HbA1C might be associated with its measurement in a limited number of women, routine screening and insurance cover of HbA1C measurements need to be considered in pregnant women and/or further studies of the criteria for pregnant women who require HbA1C measurement are required.

Several studies have attempted to use traditional statistical methods to predict GDM, based on risk factors^33,34^, but such models have not yet been employed clinically. In recent years, there have been attempts to achieve the same goal using cutting-edge, machine learning technology^22,36^. Some studies, like this one, have focused on predicting GDM using early pregnancy information, and the predictive power has been reflected by an AUC of 0.7–0.9^37–41^. Although these studies included subjects from various racial backgrounds, including Asians, there was a limited number of Asian participants, and specific studies focusing on Asian women, particularly Korean women, were scarce. Therefore, the current study is significant, as GDM varies greatly across ethnicities and geographical regions, and is particularly influenced by specific regional environments^41^. Some studies have evaluated additional biomarkers^42,43^ and genetics^44–46^, in addition to the routine data collected during antenatal care. In this study, only the basic information and laboratory tests collected during routine antenatal care were used as variables, yet the machine learning models still demonstrated moderate to high performance in early and mid-pregnancy, and have the advantage of being cost-effective.

### Strengths and limitations

Since machine learning methods are continuously evolving, numerous types of new machine learning algorithms are being developed. In this study, we used two gradient boosting algorithms. The LGBM has demonstrated good performance with bioinformatics tasks^47,48^, and XGBoost is fast to run and scalable, allows parallel computing, and solves many scientific problems accurately^27,28,49^. Following the development of a prediction model using the XGBoost algorithm with all variables included, we extracted the variables with high SHAP values or identified by the Boruta algorithm. The final simplified prediction model, which utilizes the key variables identified by the Boruta algorithm, may be more clinically practical.

One limitation is that this is a retrospective study and we conducted internal validation only, in the test set. For this reason, some data are missing and there may be bias because some tests were not performed in all populations. However, this study is based on data collected from seven centers that use the same electronic health record system and that are located in various geographical locations in Korea. Thus, our study has the strength of including various regional characteristics. In order to overcome any limitations of retrospective data, prospective cohorts for validation are currently being registered. In particular, prospective data are also being collected for the top 40 variables with high importance in SHAP value, in addition to the abbreviated variables adopted from the Boruta algorithm in this analysis. Lastly, this study evaluated the model performance not only in the whole cohort, but also in subcohorts of nulliparous and multiparous women, to improve the precision in each cohort. Our findings are predicted to be a cornerstone for developing a better algorithm and identify possible future research to validate the algorithm developed in the current study.

## Conclusion

On the basis of our study, we propose a machine learning algorithm for reliably predicting GDM in Asian women, especially Korean women. Indeed, using a machine learning model to analyze a combination of maternal factors and laboratory data may effectively predict individual risk of GDM, although further studies with prospective cohorts and external data sets may be needed for further validation.

### Supplementary Information

Supplementary Information 1.

Supplementary Information 2.

Supplementary Information 3.

Supplementary Information 4.

## Data Availability

All relevant data are within the paper and its Supporting Information files.
