# Supplementary material for: Prediction of gestational diabetes mellitus in Asian women using machine learning algorithms
Source: Sci Rep. 2023 Aug 16;13:13356. doi: 10.1038/s41598-023-39680-8 (PMC10432552; doi:10.1038/s41598-023-39680-8)
Supplement: Supplementary file 2 — Supplementary Information 2. [file 41598_2023_39680_MOESM2_ESM.docx]

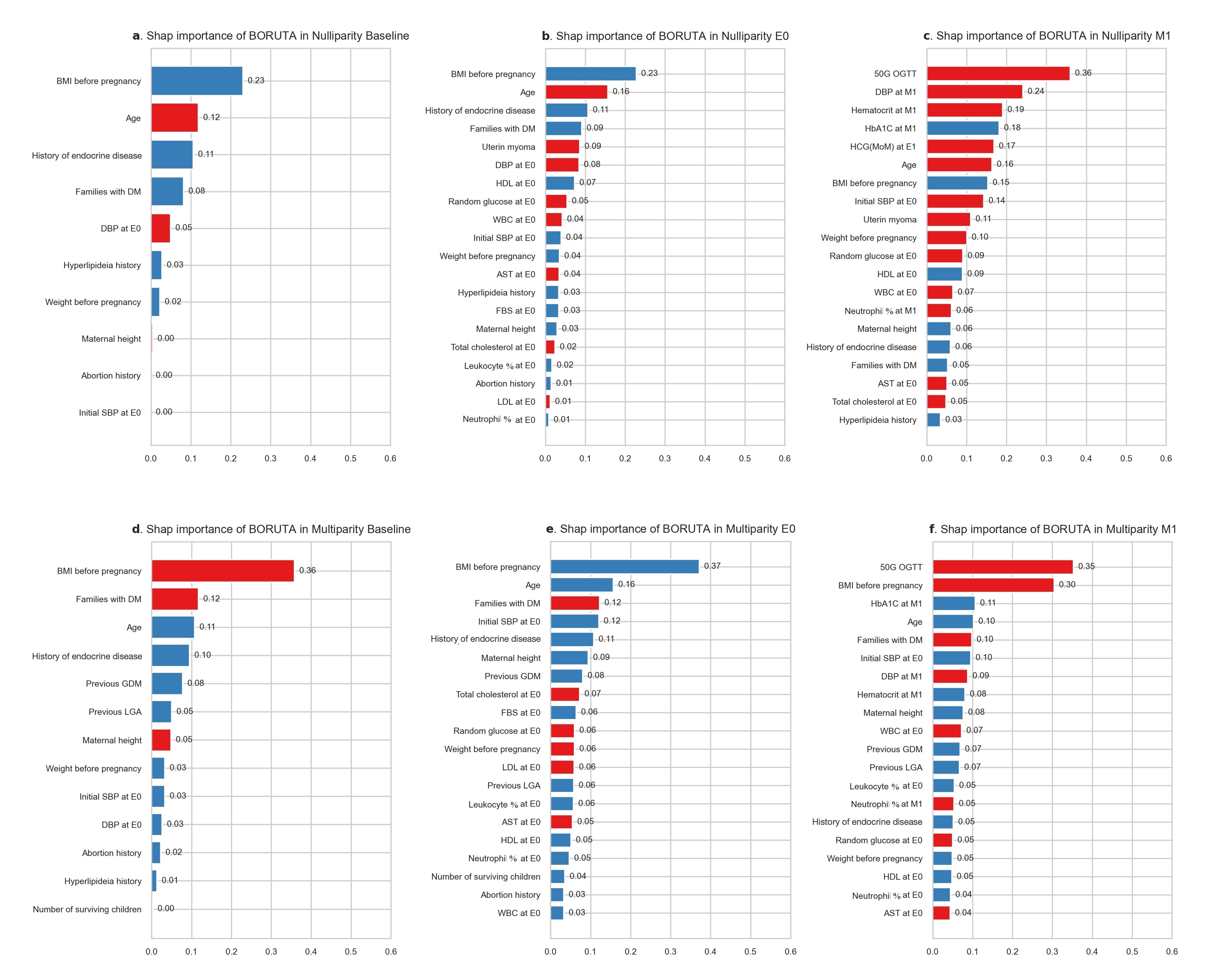


**Supplementary Fig. S2.** SHAP importance of variables identified by the Boruta algorithm in the nulliparity cohort and multiparity cohort (**a**) baseline in the nulliparity cohort, (**b**) E0 period in the nulliparity cohort, (**c**) M1 period in the nulliparity cohort (**d**) baseline in the multiparity cohort, (**e**) E0 period in the multiparity cohort, (**f**) M1 period in the multiparity cohort.
BMI, body mass index (kg/m^2^); DM, diabetes; GDM, gestational diabetes; LGA, large for gestational age; SBP, systolic BP; DBP, diastolic BP; WBC, white blood cell; HDL, high density lipoprotein; ALT, alanine aminotransferase; AST, aspartate aminotransferase; OGTT, oral glucose tolerance test; HbA1C, glycated hemoglobin; FBS, fasting blood sugar; HCG, multiples of median values of human chorionic gonadotropin; MAP, mean arterial pressure
