# Supplementary material for: Prediction of gestational diabetes mellitus in Asian women using machine learning algorithms
Source: Sci Rep. 2023 Aug 16;13:13356. doi: 10.1038/s41598-023-39680-8 (PMC10432552; doi:10.1038/s41598-023-39680-8)
Supplement: Supplementary file 3 — Supplementary Information 3. [file 41598_2023_39680_MOESM3_ESM.docx]

| **Whole** | | | | **Multiparity** | | | | **Nulliparity** | | | |
| --- | --- | --- | --- | --- | --- | --- | --- | --- | --- | --- | --- |
| **baseline** | | | | | | | | | | | |
|  | **feature** | **min** | **max** |  | **feature** | **min** | **max** |  | **feature** | **min** | **max** |
| **0** | Age | -1.066 | 0.694 | **0** | Age | -1.485 | 1.440 | **0** | Age | -1.406 | 1.001 |
| **1** | Abortion history | -0.088 | 0.737 | **1** | BMI before pregnancy | -0.929 | 1.991 | **1** | BMI before pregnancy | -0.939 | 1.721 |
| **2** | BMI before pregnancy | -0.591 | 1.585 | **2** | Families with DM | -0.251 | 1.367 | **2** | Families with DM | -0.171 | 1.330 |
| **3** | Families with DM | -0.070 | 1.092 | **3** | Maternal height | -1.536 | 0.856 | **3** | History of endocrine disease | -0.116 | 1.248 |
| **4** | Maternal height | -0.686 | 0.647 | **4** | History of endocrine disease | -0.132 | 1.349 | **4** | DBP at E0 | -1.196 | 0.764 |
| **5** | History of endocrine disease | -0.047 | 1.124 | **5** | Previous GDM | -0.059 | 3.287 | **5** | Weight before pregnancy | -0.386 | 0.470 |
| **6** | Hyperlipideia history | 0.000 | 1.404 | **6** | Initial SBP at E0 | -1.083 | 1.104 |  |  |  |  |
| **7** | Previous GDM | -0.420 | 2.491 |  |  |  |  |  |  |  |  |
| **8** | Previous LGA | -0.159 | 0.573 |  |  |  |  |  |  |  |  |
| **9** | Initial SBP at E0 | -0.777 | 0.304 |  |  |  |  |  |  |  |  |
| **E0** | | | | | | | | | | | |
|  | **feature** | **min** | **max** |  | **feature** | **min** | **max** |  | **feature** | **min** | **max** |
| **0** | Age | -0.479 | 0.541 | **0** | Age | -0.479 | 0.541 | **0** | Age | -0.820 | 0.576 |
| **1** | Abortion history | -0.020 | 0.590 | **1** | Abortion history | -0.020 | 0.590 | **1** | BMI before pregnancy | -0.375 | 1.170 |
| **2** | AST at E0 | -0.551 | 0.235 | **2** | AST at E0 | -0.551 | 0.235 | **2** | Families with DM | -0.115 | 0.751 |
| **3** | BMI before pregnancy | -0.447 | 1.112 | **3** | BMI before pregnancy | -0.447 | 1.112 | **3** | Random glucose at E0 | -0.877 | 1.661 |
| **4** | Families with DM | -0.122 | 0.784 | **4** | Families with DM | -0.122 | 0.784 | **4** | HDL at E0 | -0.040 | 0.810 |
| **5** | HDL at E0 | -0.016 | 0.749 | **5** | HDL at E0 | -0.016 | 0.749 | **5** | Uterin myoma | -0.630 | 0.067 |
| **6** | Maternal height | -0.304 | 0.144 | **6** | Maternal height | -0.304 | 0.144 | **6** | History of endocrine disease | -0.072 | 0.705 |
| **7** | History of endocrine disease | -0.063 | 0.713 | **7** | History of endocrine disease | -0.063 | 0.713 | **7** | Hyperlipideia history | -0.016 | 1.123 |
| **8** | Previous GDM | -0.040 | 2.188 | **8** | Previous GDM | -0.040 | 2.188 | **8** | DBP at E0 | -0.552 | 0.330 |
| **9** | Previous LGA | -0.049 | 0.692 | **9** | Previous LGA | -0.049 | 0.692 | **9** | Weight before pregnancy | -0.174 | 0.189 |
| **10** | Lymphocyt % at E0 | -0.056 | 0.730 | **10** | Lymphocyt % at E0 | -0.056 | 0.730 |  |  |  |  |
| **11** | Number of surviving children | -0.342 | 0.055 | **11** | FBS at E0 | -0.061 | 1.040 |  |  |  |  |
| **12** | Total cholesterol at E0 | -0.596 | 0.178 |  |  |  |  |  |  |  |  |
| **13** | Neutrophil % at E0 | -0.378 | 0.467 |  |  |  |  |  |  |  |  |
| **14** | WBC at E0 | -0.567 | 0.446 |  |  |  |  |  |  |  |  |
| **15** | FBS at E0 | -0.064 | 0.986 |  |  |  |  |  |  |  |  |
| **M1** | | | | | | | | | | | |
|  | **feature** | **min** | **max** |  | **feature** | **min** | **max** |  | **feature** | **min** | **max** |
| **0** | Age | -1.798 | 0.805 | **0** | Age | -0.949 | 0.437 | **0** | Age | -2.490 | 1.269 |
| **1** | BMI before pregnancy | -0.787 | 1.843 | **1** | BMI before pregnancy | -0.786 | 1.454 | **1** | BMI before pregnancy | -1.441 | 2.411 |
| **2** | DBP at M1 | -1.691 | 0.418 | **2** | Families with DM | -0.128 | 0.859 | **2** | DBP at M1 | -1.802 | 0.993 |
| **3** | HCG(MoM) at E1 | -1.597 | 1.145 | **3** | HbA1C at M1 | -0.088 | 3.915 | **3** | HCG(MoM) at E1 | -2.008 | 1.244 |
| **4** | Families with DM | -0.051 | 0.984 | **4** | History of endocrine disease | -0.043 | 0.503 | **4** | Families with DM | -0.235 | 0.965 |
| **5** | HbA1C at M1 | -0.063 | 4.595 | **5** | Previous GDM | -0.036 | 2.201 | **5** | HbA1C at M1 | -0.114 | 5.054 |
| **6** | Hematocrit at M1 | -1.537 | 1.109 | **6** | Previous LGA | -0.128 | 0.783 | **6** | Hematocrit at M1 | -0.992 | 1.103 |
| **7** | Uterin myoma | -1.258 | 1.126 | **7** | Initial SBP at E0 | -2.653 | 0.424 | **7** | Uterin myoma | -1.010 | 0.325 |
| **8** | History of endocrine disease | -0.025 | 0.811 | **8** | Lymphocyt % at E0 | -0.204 | 1.302 | **8** | Initial SBP at E0 | -2.098 | 0.740 |
| **9** | Previous GDM | -0.492 | 1.987 | **9** | 50G OGTT | -1.692 | 3.940 | **9** | 50G OGTT | -1.597 | 4.227 |
| **10** | Initial SBP at E0 | -1.926 | 0.964 |  |  |  |  |  |  |  |  |
| **11** | Neutrophil count at M1 | -1.828 | 1.990 |  |  |  |  |  |  |  |  |
| **12** | 50G OGTT | -2.331 | 5.877 |  |  |  |  |  |  |  |  |

**Supplementary Table. S3.** Minimum and maximum of SHAP importance of variables identified by the Boruta algorithm

BMI, body mass index (kg/m^2^); DM, diabetes; GDM, gestational diabetes; LGA, large for gestational age; SBP, systolic BP; DBP, diastolic BP; WBC, white blood cell; HDL, high density lipoprotein; ALT, alanine aminotransferase; AST, aspartate aminotransferase; OGTT, oral glucose tolerance test; HbA1C, glycated hemoglobin; FBS, fasting blood sugar; HCG, multiples of median values of human chorionic gonadotropin; MAP, mean arterial pressure
