# Supplementary material for: Prediction of gestational diabetes mellitus in Asian women using machine learning algorithms
Source: Sci Rep. 2023 Aug 16;13:13356. doi: 10.1038/s41598-023-39680-8 (PMC10432552; doi:10.1038/s41598-023-39680-8)
Supplement: Supplementary file 4 — Supplementary Information 4. [file 41598_2023_39680_MOESM4_ESM.docx]

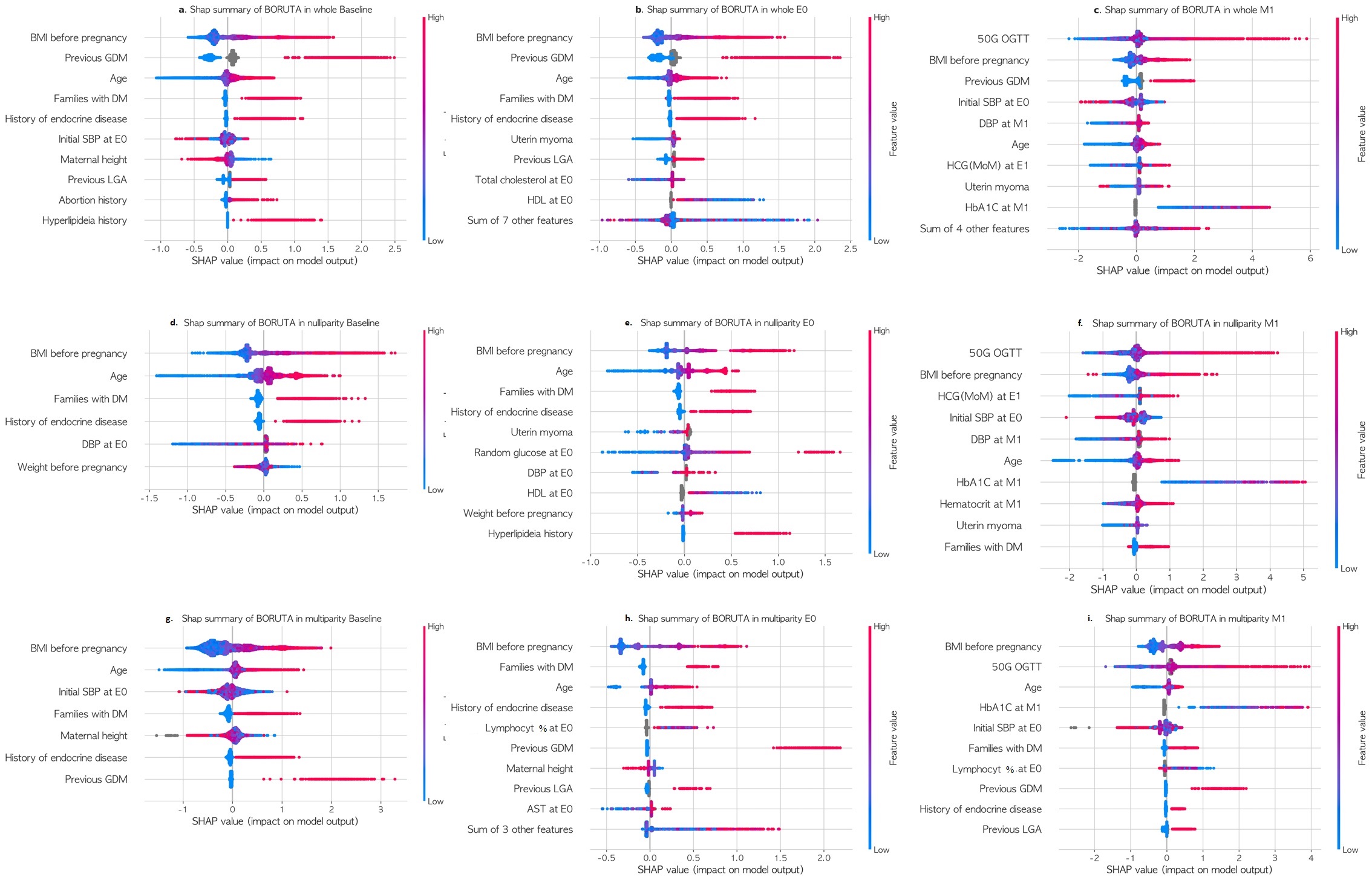


**Supplementary Fig. S4.** SHAP summary plot importance of variables identified by the Boruta algorithm (**a**) baseline in the whole cohort, (**b**) E0 period in the whole cohort, (**c**) M1 period in the whole cohort (**d**) baseline in the nulliparity cohort, (**e**) E0 period in the nulliparity cohort, (f) M1 period in the nulliparity cohort (g) baseline in the multiparity cohort, (h) E0 period in the multiparity cohort, (i) M1 period in the multiparity cohort.
BMI, body mass index (kg/m^2^); DM, diabetes; GDM, gestational diabetes; LGA, large for gestational age; SBP, systolic BP; DBP, diastolic BP; WBC, white blood cell; HDL, high density lipoprotein; ALT, alanine aminotransferase; AST, aspartate aminotransferase; OGTT, oral glucose tolerance test; HbA1C, glycated hemoglobin; FBS, fasting blood sugar; HCG, multiples of median values of human chorionic gonadotropin; MAP, mean arterial pressure
